# Theoretical and Experimental Studies on Sensitivity and Bandwidth of Thickness-Mode Driving Hydrophone Utilizing A 2-2 Piezoelectric Single Crystal Composite

**DOI:** 10.3390/s23073445

**Published:** 2023-03-24

**Authors:** Yub Je, Minseop Sim, Yohan Cho, Sang-Goo Lee, Hee-Seon Seo

**Affiliations:** 1 Agency for Defense Development, Changwon 51678, Republic of Korea; sideze@add.re.kr (Y.J.);; 2 Ibule Photonics, Incheon 21999, Republic of Korea

**Keywords:** 2-2 piezoelectric composite, single crystals, PIN-PMN-PT, thickness-mode hydrophone, broadband hydrophone

## Abstract

Piezoelectric composites, which consist of a piezoelectric material and a polymer, have been extensively studied for the applications of underwater sonar sensors and medical diagnostic ultrasonic transducers. Acoustic sensors utilizing piezoelectric composites can have a high sensitivity and wide bandwidth because of their high piezoelectric coefficient and low acoustic impedance compared to single-phase piezoelectric materials. In this study, a thickness-mode driving hydrophone utilizing a 2-2 piezoelectric single crystal composite was examined. From the theoretical and numerical analysis, material properties that determine the bandwidth and sensitivity of the thickness-mode piezoelectric plate were derived, and the voltage sensitivity of piezoelectric plates with various configurations was compared. It was shown that the 2-2 composite with [011] poled single crystals and epoxy polymers can provide high sensitivity and wide bandwidth when used for hydrophones with a thickness resonance mode. The hydrophone element was designed and fabricated to have a thickness mode at a frequency around 220 kHz by attaching a composite plate of quarter-wavelength thickness to a hard baffle. The fabricated hydrophone demonstrated an open circuit voltage sensitivity of more than −180 dB re 1 V/μPa at the resonance frequency and a −3 dB bandwidth of more than 55 kHz. The theoretical and experimental studies show that the 2-2 single crystal composite can have a high sensitivity and wide bandwidth compared to other configurations of piezoelectric elements when they are used for thickness-mode hydrophones.

## 1. Introduction

High-frequency sonars have been receiving attention along with the recent developments of small platforms such as Unmanned Undersea Vehicles (UUVs) and Unmanned Surface Vehicles (USVs). These sonars operate at frequencies above 100 kHz to achieve a spatial resolution of centimeters and detection range of hundreds of meters. Many industries are developing various types of high-frequency imaging sonars, such as Side Scan Sonars (SSS), Synthetic Aperture Sonars (SAS), mine-hunting sonars, diver-held sonars, and their intercepting sonars [1,2,3,4,5,6,7,8]. The hydrophones of these high-frequency imaging sonars are generally made into a planar array attached to the surface of the platform to create the required array beams. The hydrophone element is designed to have a high sensitivity and wide bandwidth to improve detection range and temporal resolution.

Hydrophones detect the pressure variations of underwater acoustic signals and convert them into an output voltage. One of the hydrophone response modes is the hydrostatic mode [9,10]. Hydrophones with the hydrostatic mode are normally operated far below their resonance frequency. Their response is flat over a wide frequency band, but the sensitivity level is relatively low. The wavelength is large enough compared to the hydrophone dimensions to ensure uniform pressure over the entire hydrophone surface. Cylindrical and spherical piezoelectric hydrophones are widely used for hydrostatic mode hydrophones. They have good pressure sensitivity together with a robust structure under hydrostatic pressure. Another response mode of the hydrophone, the resonance mode, is normally operated near their resonance frequency [9,11]. Their sensitivity level is relatively high, but the response is non-flat in the operating frequency band. The wavelength is comparable to the hydrophone dimensions, so the acoustic pressure mainly acts on only the acoustic surfaces. Piezoelectric plates driven with their thickness mode are suitable for high-frequency sonars because it is easy to construct a planar array that can have high sensitivity. Single-phase piezoelectric materials or piezo-material/polymer composites can be used for this type of hydrophone.

Piezoelectric composites utilizing piezoelectric materials and a polymer matrix offer advantages over single-phase piezoelectric materials [9]. A principal advantage of piezoelectric composites is that the material properties, such as density, stiffness, acoustic impedance, and piezoelectric properties, can be controlled by the volume fraction of piezoelectric materials and polymer matrix. For projectors, composites can improve the electromechanical coupling coefficient and frequency bandwidth by reducing mechanical stiffness. For hydrophones, composites can improve the bandwidth by reducing their acoustic impedance close to the medium and improve the sensitivity with a large piezoelectric constant. The flexibility of the polymer matrix also enables a hydrophone array conforming to the platform shape. The piezoelectric composites with 1-3 connectivity and 2-2 connectivity are common because of their large electromechanical coupling and fabrication simplicity [9,10,11,12,13,14].

PbZrTiO_3_ (PZT) ceramics, which offer relatively high piezoelectric coefficients (d~590 pC/N) and electromechanical coupling factors (k_33_~0.75), are the most widely used active material for piezoelectric composites. Since PZT ceramic composites can be fabricated cost-effectively by the injection molding process [15], acoustic sensors based on PZT ceramic composites are successfully commercialized. These 1-3 PZT composites or single-phase PZT plates have thus been used for acoustic sensors in most current developing high-frequency sonars [3,4,5,6,7,8]. With increasing interest in high-resolution imaging sonars, acoustic sensors with increased bandwidth and sensitivity are being demanded [5,6,16,17].

In recent studies [18,19,20,21,22,23,24,25,26], relaxor-PT single crystals such as Pb(Mg_1/3_Nb_2/3_)O_3_-PbTiO_3_ (PMN-PT) and Pb(Zn_1/3_Nb_2/3_)O_3_-PbTiO_3_ (PZN-PT) have been investigated as active materials for composites. Single crystals exhibit superior piezoelectric properties compared to PZT ceramics; for example, they have piezoelectric coefficients d over 1500 pC/N and electromechanical coupling factors k_33_ around 0.90 [27,28,29]. Therefore, it is expected that single crystals can further improve the sensitivity, source level, and frequency bandwidth when used as the active material of the composite. Furthermore, the superior hydrostatic figure of merit (d_h_g_h_) of single crystal composites can improve the signal-to-noise ratio of hydrophones with the hydrostatic mode. Single crystal composites with 1-3 connectivity have been mainly studied for acoustic sensors [18,19,20]. However, their fabrication process, the dice-and-fill process, requires attention and optimization due to the possible fracture and internal stress within the single crystals. Lili et al. [21] reported that 2-2 single crystal composites, which are lamellar stacks of [011] poled PMN-PT single crystal layers and polymer layers show superior hydrostatic Figure of Merit (FoM) over 1-3 single crystal composites. Even though 2-2 composites show slightly lower electromechanical coupling compared to 1-3 composites, they have merits associated with the dice-and-fill fabrication process. Several further studies [22,23,24,25,26] investigated 2-2 composites based on single crystals for high sensitivity and wide bandwidth.

In this study, a 2-2 piezoelectric single crystal composite was studied for use as a hydrophone with a thickness resonance mode. From the theoretical analysis, material properties that determine bandwidth and sensitivity of the thickness-mode piezoelectric plate were derived. The material properties and the voltage sensitivity of piezoelectric plates with various configurations were calculated and compared by finite element analysis. It is shown that the 2-2 composite with [011] poled single crystals and epoxy polymers can provide high sensitivity and wide bandwidth when used for hydrophones with a thickness resonance mode. The hydrophone element was designed and fabricated to have a thickness mode at a frequency of around 220 kHz. The Pb(In_1/2_Nb_1/2_)O_3_-Pb(Mg_1/3_Nb_2/3_)O_3_-PbTiO_3_ (PIN-PMN-PT) single crystal grown by the Bridgman method was fabricated into a 2-2 composite using the dice-and-fill process. To determine the resonance, a composite plate of quarter-wavelength thickness was attached to a hard baffle that can withstand the hydrostatic pressure of a deep-sea operation. The fabricated composite and hydrophone were tested to validate their electric and acoustic characteristics.

## 2. Theory and Simulation

In this section, theoretical and numerical analysis of the piezoelectric plate are described. The material properties that determine the voltage sensitivity and the bandwidth of the piezoelectric plate are derived from theoretical analysis. Then, various piezoelectric plates with different configurations and materials are suggested and compared by numerical analysis.

### 2.1. Theoretical Analysis of the Piezoelectric Plate

Piezoelectric plates with large lateral dimensions compared to their thickness can be considered for thickness-mode hydrophones. The laterally clamped condition of the plate increases the stiffness and the wave speed in the thickness direction. The plates can be backed by a pressure release material to operate in half-wavelength thickness mode or backed by a hard backing plate to operate in quarter-wavelength thickness mode. In this study, a single crystal composite backed by a hard backing plate was considered for the deep-depth operation of the hydrophone.

**Figure 1:**
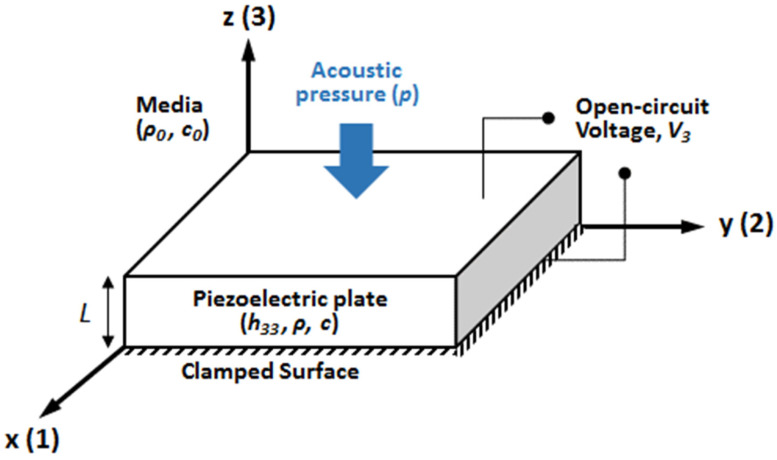
Piezoelectric plate for the thickness-mode hydrophone. The plate with a thickness *L* with a clamped bottom surface and electrically open circuit.

Figure 1 shows a piezoelectric plate of a thickness *L* with a clamped bottom surface. The plate is poled in the thickness direction 3. The constitutive equations of interest for the piezoelectric plate are as follows [30]:

(1) $$\begin{array}{c} T=c^{D}S-h_{33}D\\ E=-h_{33}S+\beta^{S}D \end{array}$$

where the equations relate mechanical stress *T*, mechanical strain *S*, electric field *E*, and electric displacement *D*. $c^{D}$ is mechanical stiffness, $h_{33}$ is the piezoelectric constant, and β is the impermittivity constant. The top and bottom electrodes are under an open circuit condition ($D_{3}=0)$. The lateral clamping conditions ($S_{1}=S_{2}=S_{4}=S_{5}=S_{6}=0)$ and electric flux leakage-free conditions ($D_{1}=D_{2}=\partial D_{3}/\partial z=0)$ of the plate suggest the constitutive equations:

(2) $$\begin{array}{c} T_{3}=c_{33}^{D}S_{3}\\ E_{3}=-h_{33}S_{3} \end{array}$$

The displacement field ζ in the thickness direction z of the piezoelectric plate is

(3) $$\zeta \left(z\right)=B\cos kz+D\sin kz$$

where *k* = *ω*/*c* is the wave number. The boundary condition of the clamped base ($\zeta \left(0\right)=0)$ suggests B=0. The wave speed c of the piezoelectric plate can be obtained by using the relationship $\partial T/\partial z=\rho \partial^{2}\zeta /\partial t^{2}$ [30] as follows:

(4) $$c=\sqrt{\frac{c_{33}^{D}}{\rho }}$$

The mechanical impedance $Z_{m}$ of the plate is obtained by using Equations (2)–(4):

(5) $$Z_{m}\left(z\right)=\frac{F\left(z\right)}{u\left(z\right)}=\frac{-A_{0}T_{3}}{\partial \zeta /\partial t}=\frac{-A_{0}c_{33}^{D}\partial \zeta /\partial z}{\partial \zeta /\partial t}=\frac{-A_{0}c_{33}^{D}k\cos kz}{j\omega \sin kz}=j\rho cA_{0}\cot kz$$

where $A_{0}$ is the surface area of the plate and u is the velocity of the plate. The radiation impedance of the plate with large lateral dimensions is $Z_{r}\approx \rho_{0}c_{0}A_{0}$ where $\rho_{0}$ and $c_{0}$ are the density and wave speed of the medium [31]. The displacement on the top surface of the plate under acoustic pressure excitation p is obtained using Equation (5):

(6) $$\zeta \left(L\right)=\frac{1}{j\omega }u\left(L\right)=\frac{1}{j\omega }\frac{F\left(L\right)}{Z_{m}\left(L\right)+Z_{R}}=\frac{1}{j\omega }\frac{-A_{0}p}{j\rho cA_{0}\cot kL+\rho_{0}c_{0}A_{0}}$$

The voltage across the electrode is obtained using Equations (2) and (6):

(7) $$V_{3}=\int_{0}^{L}E_{3}dz=\int_{0}^{L}-h_{33}\frac{\partial \zeta }{\partial z}dz=-h_{33}\zeta \left(L\right)=\frac{h_{33}}{j\rho_{0}c_{0}\omega }\frac{1}{1+j\rho c/\rho_{0}c_{0}\cot kL}p$$

The voltage sensitivity of the piezoelectric plate is

(8) $$G=V_{3}/p=\frac{h_{33}}{j\rho_{0}c_{0}\omega }\frac{1}{1+j\rho c/\rho_{0}c_{0}\cot kL}$$

Because the piezoelectric plate has the thickness of a quarter-wavelength ($L=\lambda /4=c\pi /2\omega_{0}$) at the resonance frequency $\omega_{0}$, the voltage sensitivity of the piezoelectric plate can be expressed as

(9) $$G=\frac{h_{33}}{j\rho_{0}c_{0}\omega }\frac{1}{1+j\rho c/\rho_{0}c_{0}\cot\frac{\pi }{2}\frac{\omega }{\omega_{0}}}$$

Equation (9) indicates that the voltage sensitivity of a piezoelectric plate is related to the material properties of the piezoelectric constant, $h_{33}$, and the acoustic impedance ρc. Near the thickness resonance frequency $\omega =\omega_{0}$, the sensitivity approaches its maximum value of $h_{33}/j\rho_{0}c_{0}\omega_{0}$ as the term of the cotangent function approaches zero. Therefore, single crystals with a high piezoelectric constant, $h_{33}$, are advantageous in terms of sensitivity. In terms of bandwidth, it can be increased when the sensitivity changes slowly with frequency. From Equation (9) the term of the cotangent function dominates the change of sensitivity near the resonance frequency. Therefore, wide bandwidth can be achieved when the acoustic impedance of the plate, ρc, is decreased close to the acoustic impedance of the medium $\rho_{0}c_{0}$. The composites that allow control of their acoustic impedance to a lower value can have a much larger bandwidth than single-phase materials.

### 2.2. Piezoelectric Plates for Thickness-Mode Hydrophones

This study suggests and compares different configurations of piezoelectric plates that can be used for thickness-mode hydrophones based on finite element analysis. Table 1 lists the suggested configurations of the piezoelectric plates, which include PZT-5H ceramic, [001] poled PIN-PMN-PT single crystal, and [011] poled PIN-PMN-PT single crystal with configurations of single phase, 1-3 composite, and 2-2 composite. PZT ceramics and [001] poled single crystals are suggested for active materials of the 1-3 composite in this study. They have the highest piezoelectric constant in the thickness direction 3. The 1-3 composites with these active materials also have an advantage because the polymer matrix can reduce the destructive interference from their negative piezoelectric coefficients in the lateral directions 1 and 2. For active materials of 2-2 composite, [011] poled single crystals are suggested. The [011] poled single crystal exhibits positive piezoelectric constants in the 1 and 3 directions, while it has negative piezoelectric constants in the 2 direction [18]. The 2-2 composites have an advantage in their configuration because the polymer layers, which are perpendicular to the 2 direction, reduce the destructive interference from the negative piezoelectric coefficient in the 2 direction.

The material properties and the voltage sensitivity of the piezoelectric plates in Table 1 are simulated by using the commercial finite element analysis tool, COMSOL multiphysics. All the piezoelectric plates in the finite element model have lateral dimensions of 10 mm × 10 mm and a thickness of a quarter wavelength at the resonance frequency, *f_r_*. The bottom surfaces of the plates are clamped, and top and bottom electrodes are electrically open. The composite plates have a volume fraction of active material of 0.3 with the matrix material of Epotek 301 epoxy [32]. A quasi-static stress, or plane acoustic wave from water medium were introduced to the top surface of the piezoelectric plates to calculate the related material properties, or voltage sensitivity, respectively.

As discussed in the previous section, it is shown that the material properties of piezoelectric constant and the acoustic impedance dominate the characteristics of piezoelectric plates. In this analysis, the material properties of the suggested piezoelectric plates were obtained from finite element analysis because the theoretical derivation of equivalent material properties of the composites requires complicated formulations. The induced strain *S_3_* and electric field *E_3_* under quasi-static stress *T_3_* were calculated using the finite element model. Then, the material properties of $h_{33}$ and $c_{33}^{D}$ were derived using the stress–strain relation and the field–strain relation in Equation (1). Table 2 lists derived material properties of the piezoelectric plates.

Among the single phase plates, the single crystal plate shows a superior piezoelectric constant $h_{33}$ compared to the PZT ceramic plate. However, their higher acoustic impedance compared to that of the medium (~1.5 MRayls) results in a narrow bandwidth in their sensitivity. The composite plate shows a higher piezoelectric constant $h_{33}$ and much lower acoustic impedance compared to the single-phase plate. The configurations of composite plates, in which active material pillars with a high aspect ratio are surrounded by an epoxy matrix, can greatly reduce the lateral clamping stiffness of the plate. Therefore, the sound speed of the composite plate can be reduced along with their density. The 2-2 composite based on [011] poled PIN-PMN-PT single crystal is shown to have highest piezoelectric constant and sufficiently low acoustic impedance.

**Figure 2:**
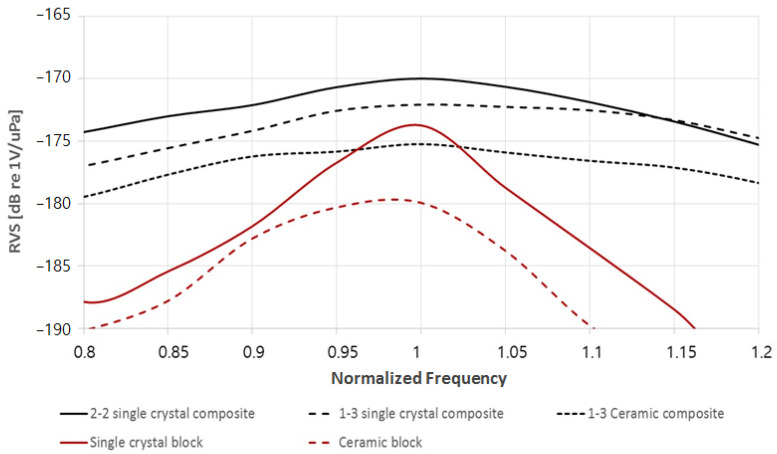
The simulated receiving voltage sensitivity of piezoelectric plates: 2-2 single crystal composite (black solid line), 1-3 single crystal composite (black dashed line), 1-3 ceramic composite (black dotted line), single crystal block (red solid line), and PZT ceramic with single phase (red dashed line).

Figure 2 shows the receiving voltage sensitivity of the suggested piezoelectric plates, calculated using the finite element analysis. All the plates are modeled to have the same thickness-mode resonance frequency *f_r_*, and the frequency scale is normalized to the resonance frequency. The sensitivity shows a maximum at the resonance frequency in the simulated curves. As expected, the composites show much broader bandwidth and higher sensitivity than the single-phase materials because of their low acoustic impedances and high piezoelectric constants. Single crystals offer higher sensitivity than PZT ceramics when they are used for both single phase plates and composite plates. The simulation results show that the 2-2 composite based on [011] poled PIN-PMN-PT single crystal can have high sensitivity with a broad bandwidth when used for thickness-mode hydrophones. Considering the fabrication simplicity of the 2-2 composite compared to the 1-3 composite, the 2-2 single crystal composites fabricated by the dice-and-fill process have advantages in both performance and manufacturability.

## 3. Design and Fabrication

The thickness-mode driving hydrophone, based on the 2-2 single crystal composite, was designed and fabricated according to the analysis in Section 2. The PIN-PMN-PT single crystal grown by the Bridgman method was fabricated into a 2-2 composite by the dice-and-fill process. The composite with quarter-wavelength thickness was attached to a hard baffle in order to have a thickness mode at the operating frequency.

**Figure 3:**
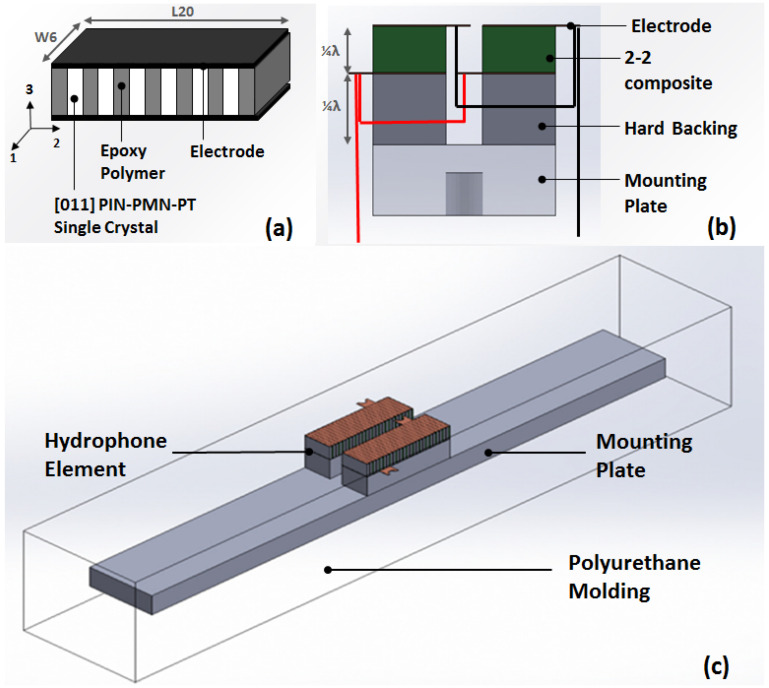
The schematic drawing of the hydrophone based on the 2-2 single crystal composite: (**a**) The single crystal-polymer composite with 2-2 connectivity. (**b**) The hydrophone element utilizing the 2-2 composite with a hard backing. (**c**) The overall hydrophone.

Figure 3 shows a schematic design of the hydrophone. The 2-2 composite is a lamellar stack of the single crystal layers and the polymer layers, as shown in Figure 3a. The PIN-PMN-PT crystal poled in [011] crystal direction and Epotek 301 epoxy are used for active material and polymer material, respectively. The length and width of the composite are 20 mm and 6 mm, respectively. The lateral dimensions were determined to have the required element acoustic beam without lateral vibration modes at the operating frequency. The thickness of the composite is 2.75 mm, which was adjusted using FEM analysis to have a quarter-wavelength resonance at 220 kHz. The volume fraction of the active material in the composite is 0.35 and the kerf size (the width of the polymer layer) is 500 μm. The electrode plates are attached on the top and bottom surface of the composite.

Figure 3b shows the hydrophone element utilizing the 2-2 composite with a hard backing. For the hard backing, we choose a stainless steel block, which has sufficiently high acoustic impedance compared to that of the composite. By attaching the quarter-wavelength thickness composite on the hard backing plate, the composite can operate in its thickness mode at the designed frequency. Two elements are connected in parallel as one channel to secure the required capacitance. The hydrophone elements are aligned on the polycarbonate mounting plate and waterproofed using polyurethane molding, as shown in Figure 3c.

**Figure 4:**
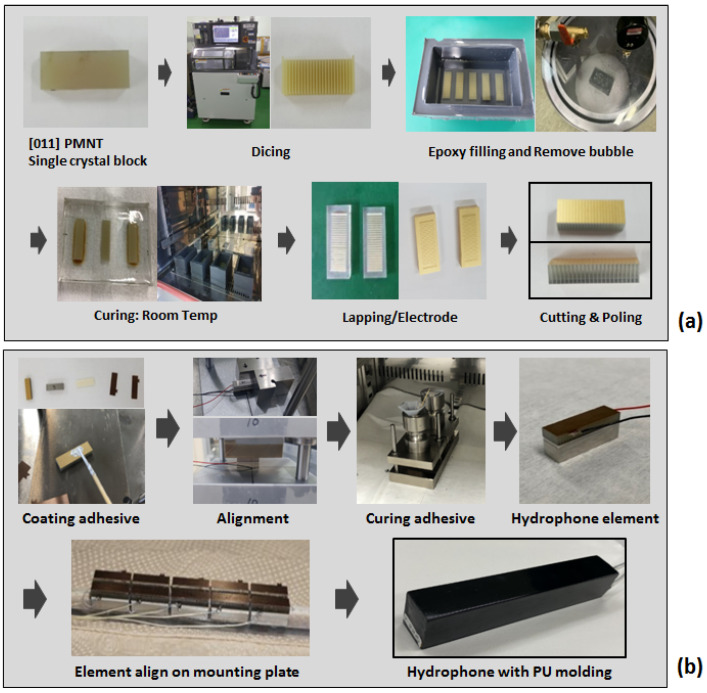
Fabrication process: (**a**) The 2-2 single crystal composite. (**b**) The hydrophone.

Figure 4 shows the fabrication process of the hydrophone. The PIN-PMN-PT single crystal grown by the Bridgman method was diced into the designed kerf size. Epoxy was filled in the diced kerf in a vacuum chamber and was cured at room temperature. After lapping and depositing the electrode on the top and bottom surfaces of the composite, the composite was poled and was cut into the required size. The composite, electrodes, and backing block were bonded into the hydrophone element. The elements were aligned on the mounting plate with connecting wire and then molded by the polyurethane for waterproofing.

## 4. Experimental Results and Discussions

In this section, the test results of the fabricated composite and the hydrophone are described. Electrical characteristics and acoustic characteristics of the composite and hydrophone were tested and compared with the simulated data for the verifications.

### 4.1. Electrical Test of the 2-2 Composite

**Figure 5:**
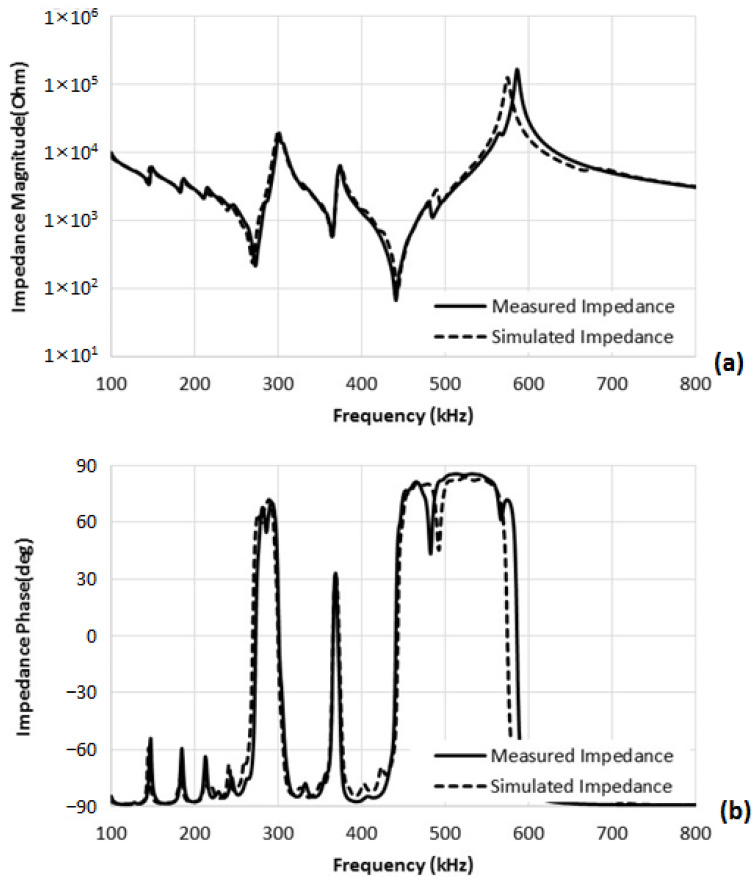
Measured (solid line) and simulated (dashed line) impedance of the 2-2 composite: (**a**) Impedance magnitude. (**b**) Impedance phase.

The fabricated composites, not attached to the hard baffle, were tested and compared with the simulated data to validate their electrical characteristics. Figure 5 shows the measured and simulated impedance curves of the 2-2 composite. The simulated curves were calculated from the finite element model of the composite. The well-matched curves of simulated and matched data show that the composites were fabricated as we designed. The measured resonance frequency and anti-resonance frequency of the thickness mode were 443 kHz and 587 kHz, respectively. The effective electromechanical coupling coefficient of the composite was 0.66. Since the composite has a free boundary condition without bottom clamping, the anti-resonance frequency of the composite does not coincide with the designed frequency. After attaching the composite to the hard baffle, the anti-resonance frequency decreases to approximately the designed frequency of 220 kHz, where the sensitivity reaches its maximum. Other resonance frequencies of 270 kHz and 364 kHz shown in the graph are lateral resonance frequencies of the composite.

### 4.2. Acoustic Test of the Hydrophone

**Figure 6:**
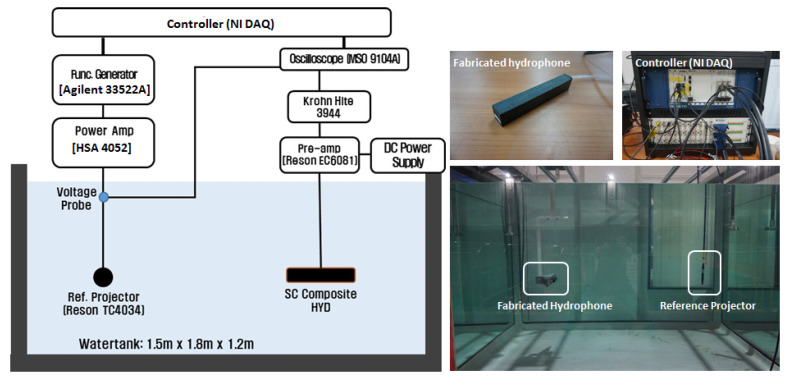
Acoustic experimental set-up of the hydrophone.

The fabricated hydrophone was tested to validate its acoustic characteristics. The open circuit voltage sensitivity and beam patterns were measured in water-tank facilities. Figure 6 shows the experimental set-up for the hydrophone. The experiments were performed in a 1.5 × 1.8 × 1.2 m water tank with motorized linear and rotation stages. The calibrated reference projector (TC4034) was used to measure the receiving voltage sensitivity of the hydrophone. The hydrophone and the reference projector were installed at the same depth of 0.5 m, with a distance of 1 m in the water tank. An input signal generated from a function generator (Agilent 33522A) was amplified through a power amplifier (HSA 4052) and fed into the reference projector to radiate sound. The measured acoustic signal from the hydrophone was pre-amplified, band-pass filtered (Krohn Hite 3944), and visualized on a digital oscilloscope and a controller (NI PCIe-8861). The voltage levels generated from the hydrophone were measured from the NI controller while changing frequencies from 120 kHz to 280 kHz in step of 5 kHz. The voltage sensitivity of the hydrophone with frequency was calculated by using the calibrated transmitting voltage response of the projector

**Figure 7:**
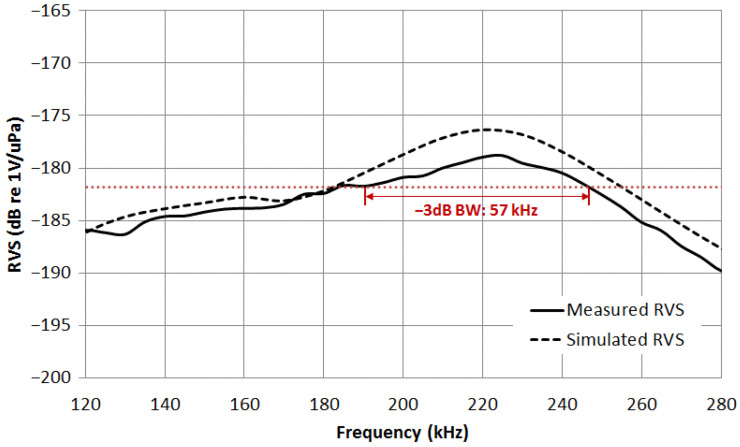
Measured (solid line) and simulated (dashed line) open circuit voltage sensitivity of the hydrophone.

Figure 7 shows the measured and the simulated open circuit voltage sensitivity curves of the hydrophone. The simulated sensitivity curve was calculated from the finite element model of the hydrophone. The maximum voltage sensitivity was −178.5 dB at the thickness-mode resonance frequency of 224 kHz, which is close to the designed frequency. The measured maximum voltage sensitivity at about 3 dB lower than the simulated one. We suspect that the fabrication conditions of the sensor may not be perfect enough. The imperfect adhesion between the composite plate and the hard backing plate may reduce the measured level of sensitivity. The −3 dB bandwidth of the hydrophone was measured to be 57 kHz, with the fractional bandwidth of 25.4%. The bandwidth is inversely proportional to the temporal resolution: resolution = sound velocity/(2 × bandwidth) [34]. The across-track resolution of state-of-the-art high frequency sonar is on the order of a few centimeters, which requires a bandwidth over 30 kHz [4,6]. The proposed hydrophone shows the more than equal bandwidth compared to the sensors in the current developing imaging sonar field.

**Figure 8:**
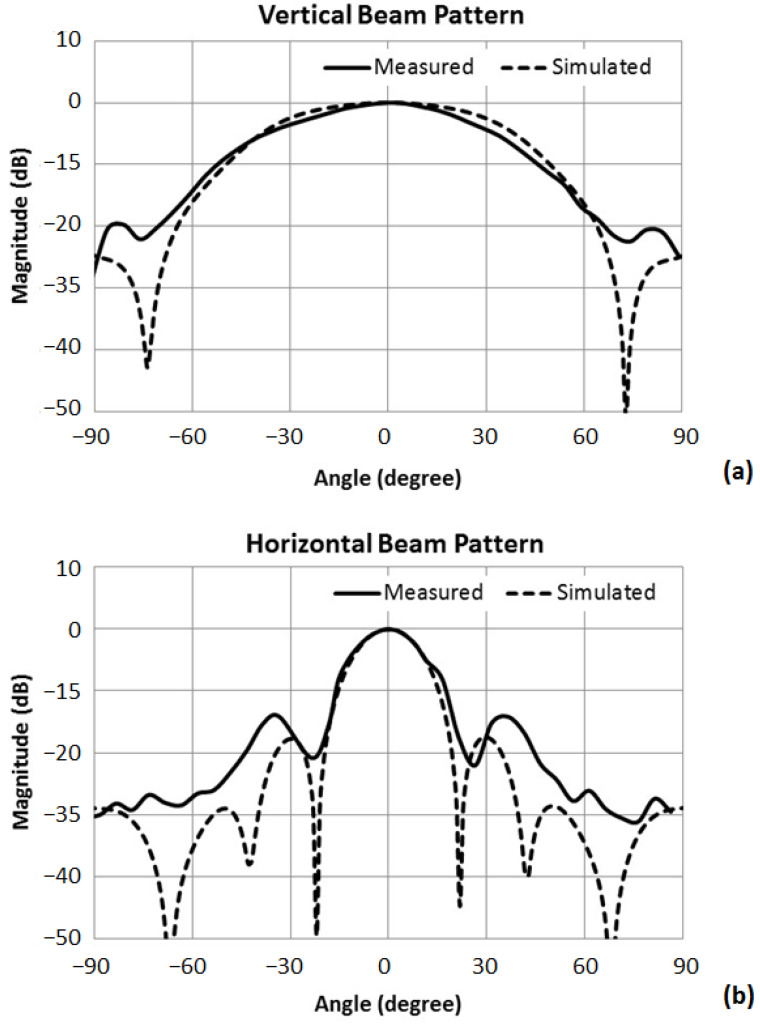
Measured (solid line) and simulated (dashed line) beam patterns of the hydrophone: (**a**) Vertical beam patterns. (**b**) Horizontal beam patterns.

Figure 8 shows the measured and simulated beam patterns of the hydrophone near the resonance frequency. The simulated beam patterns were calculated from the finite element model of the hydrophone. The vertical and horizontal beam patterns were measured using a rotating hydrophone in the water tank. The measured beam patterns were well matched to the simulated beam patterns in their major lobes. The measured and simulated beam patterns showed differences in their minor lobes. The measured voltage level was too low in their minor lobes, including null points, to achieve precise measured data. The −3 dB beam width of the vertical beam pattern and the horizontal beam pattern were 48° and 19.5°, respectively. The hydrophone element was designed to have relatively wide vertical beam width to ensure sufficient vertical coverage of the sonar. The horizontal beam was designed to be sharp enough to suppress grating lobes of the array beam. Since the beam pattern of the array is represented by the product of the beam patterns of the element and the array with point sources [8], a sharp horizontal beam of the element can suppress the grating lobe of the array, which can be a spurious signal in the sonar.

## 5. Conclusions

This study investigated a thickness-mode driving hydrophone utilizing a 2-2 piezoelectric single crystal composite. From the theoretical and numerical analysis, material properties that determine the bandwidth and sensitivity of the thickness-mode piezoelectric plate were derived, and the voltage sensitivity of piezoelectric plates with various configurations was compared. It was shown that the 2-2 composite with [011] poled single crystals and epoxy polymers can provide high sensitivity and wide bandwidth when used for hydrophones with a thickness resonance mode. The hydrophone element was designed and fabricated to have a thickness mode at a frequency around 220 kHz by attaching a composite plate of quarter-wavelength thickness to a hard baffle. The fabricated hydrophone showed a high open circuit voltage sensitivity of over −180 dB re 1 V/μPa and a wide bandwidth of over 55 kHz. The theoretical and experimental studies show that the 2-2 single crystal composite can have high sensitivity and wide bandwidth compared to other configurations of piezoelectric elements when they are used for thickness-mode hydrophones.

## Figures and Tables

**Table 1:** Configurations and active materials of the piezoelectric plates for thickness-mode hydrophones.

No	Phase	Material	Configuration
1	Single phase	PZT ceramics (PZT-5H)	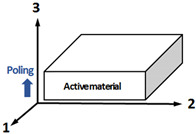
2	Single phase	Single crystal ([001] PIN-PMN-PT)	
3	1-3 composite	PZT ceramics/Epoxy (PZT-5H/Epotek 301)	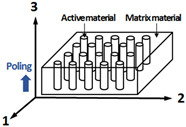
4	1-3 composite	Single crystal/Epoxy ([001] PIN-PMN-PT/Epotek 301)	
5	2-2 composite	Single crystal/Epoxy ([011] PIN-PMN-PT/Epotek 301)	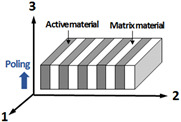

**Table 2:** Sensitivity-related material properties of the piezoelectric plates for thickness-mode hydrophones.

Piezoelectric Plates	Sensitivity Related Material Properties				
	*h*_33_ (GV/m)	$c_{33}^{D}$ (GPa)	*ρ* (kg/m^3^)	*c* ^1^ (m/s)	*Ρc* (MRals)
Single Phase (block) (PZT-5H [9])	1.78	157	7500	4580	34.3
Single Phase (block) ([001] PIN-PMN-PT [9])	2.41	160	8120	4430	36.0
1-3 composite ^2^, (PZT-5H/Epotek 301 [32])	2.15	27.0	3760	2660	10.2
1-3 composite ^2^ ([001] PIN-PMN-PT/Epotek 301)	3.06	25.9	3820	2600	9.9
2-2 composite ^2^ ([011] PIN-PMN-PT [33]/Epotek 301)	3.78	33.1	3820	2945	11.2

^1.^ Open circuit sound speed of plate is calculated from the equation: $c=\sqrt{c_{33}^{D}/\rho }.$ ^2.^ Volume fraction of active materials of the composite is 0.3.

## Data Availability

Not applicable.
